# Proactive Early Warning of Vortex Ring State in Coaxial UAVs: A Physics-Informed Multimodal ViT-LSTM Approach

**DOI:** 10.3390/s26123888

**Published:** 2026-06-18

**Authors:** Xiang Zhou, Jiawei Sun, Jiannan Zhao, Feng Shuang

**Affiliations:** Guangxi Key Laboratory of Intelligent Control and Maintenance of Power Equipment, School of Electrical Engineering, Guangxi University, No. 100, Daxue East Road, Nanning 530004, China; xiang.zhou@st.gxu.edu.cn (X.Z.); jiaweisun@st.gxu.edu.cn (J.S.)

**Keywords:** Vortex Ring State (VRS), coaxial UAVs, proactive early warning, multi-sensor fusion, Vision Transformer (ViT), Hybrid Ordinal Loss, condition monitoring

## Abstract

The Vortex Ring State (VRS) poses a catastrophic aerodynamic threat to coaxial dual-rotor unmanned aerial vehicles (UAVs). Traditional reactive detection mechanisms provide insufficient altitude for recovery, while existing data-driven diagnostics are severely bottlenecked by data leakage, extreme class imbalance, and a lack of physical interpretability. To bridge these gaps, this paper proposes a physics-informed multimodal deep learning framework that transitions from post-occurrence detection to proactive early warning. We establish a 1.5 s precursor window—creating a three-class ordinal state space—to provide the flight control system with critical intervention time for differential rotor recovery. We developed a novel ViT-LSTM architecture (MTSF-Net) to fuse continuous seven-channel onboard-recorded data (comprising three-axis acceleration, three-axis angular velocity, and barometric vertical velocity), which are subsequently transformed into Continuous Wavelet Transform (CWT) spectrograms. To ensure real-time unidirectional inference while preserving absolute physical vibration scales across heterogeneous sensors, a Calibrated Benchmark Normalization (CBN) strategy is introduced. Furthermore, a Hybrid Ordinal Loss is proposed to mitigate the extreme sample imbalance (<0.5%) of the precursor state by penalizing asymmetric aerodynamic degradation. Evaluated under a strict sortie-based isolation protocol, the proposed system achieves an exceptional test accuracy of 98.26% and an unprecedented precursor recall of 100%. Notably, it completely eliminates fatal missed detections (VRS predicted as Normal) and false-positive VRS predictions triggered by precursor states. Finally, Gradient-weighted Class Activation Mapping (Grad-CAM) is utilized to verify that the multimodal sensor processing pipeline successfully anchors onto authentic physical vibration frequencies rather than artifactual noise, laying a rigorous, interpretable foundation for intelligent aviation safety systems.

## 1. Introduction

With the rapid proliferation of unmanned aerial vehicles (UAVs) in autonomous aviation and industrial inspections, the operational safety boundaries of rotorcraft are facing unprecedented challenges. Among various aerodynamic hazards, the Vortex Ring State (VRS) remains the most critical issue due to its sudden onset and high fatality rate [1]. When a rotorcraft descends at a rate approaching its induced velocity, the downwash forms a toroidal vortex structure, leading to a precipitous drop in lift and uncontrollable oscillations [2].

Traditionally, flight control systems rely on “passive detection,” triggering alarms only after macroscopic kinematic anomalies—such as surging descent rates—are registered by barometers or GPS [3]. However, once a fully developed VRS is established, control effectiveness is drastically diminished, leaving insufficient altitude for recovery [1]. Consequently, there is an urgent industry demand for a “precursor early warning” system capable of proactive interception during the incipient phase.

Although data-driven models show promise, they encounter two critical engineering pitfalls in aviation: their inherent “black-box” nature, which hinders airworthiness certification, and the issue of “data leakage” during time-series processing, which severely degrades cross-sortie generalization [4,5]. This study proposes a physics-informed multimodal deep learning architecture to resolve these dilemmas. The contributions of this paper are as follows:

1. Establishment of a three-class ordinal early warning system. We define a 1.5 s precursor window, constructing a temporal trajectory of Normal → Precursor → VRS to grant the flight control system ample intervention time [6].
2. Proposal of the MTSF-Net architecture and the companion CBN strategy. We pioneer the novel CBN pre-processing strategy to preserve absolute physical vibration scales across sensors while ensuring real-time unidirectional inference, resolving the flaws of local normalization.
3. System Reliability Verification via Grad-CAM. We provide visually quantifiable evidence that the model’s decisions anchor onto authentic physical frequency bands, closing the loop between AI sensing and rotor aerodynamics to ensure engineering credibility [7].

## 2. Related Work

### 2.1. Traditional Rotor Dynamics and Threshold-Based Detection

**Figure 1:**
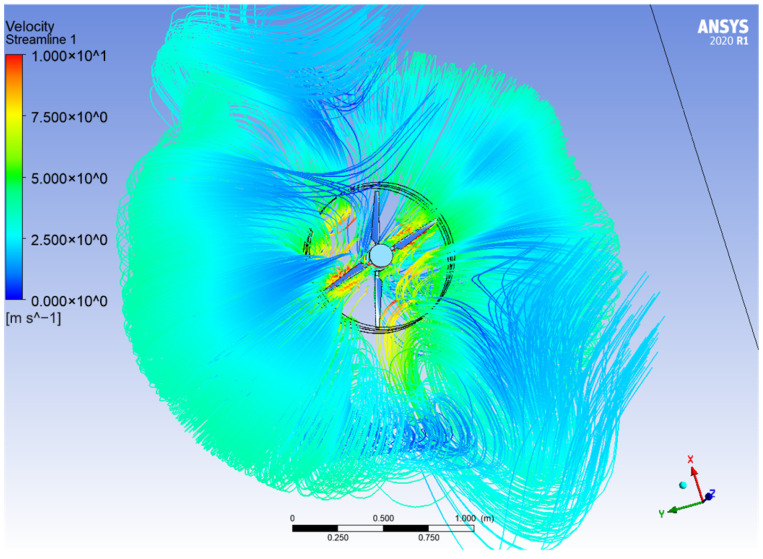
Flow field streamline distribution of coaxial dual rotor under VRS as shown by CFD simulation. It can be seen that the distribution of streamline velocity and position along the circumference of the rotor disk is extremely non-uniform, causing periodic changes in rotor aerodynamic load.

Historically, theoretical analysis of the Vortex Ring State (VRS) has relied on classical aerodynamic models, which often suffer from mathematical divergence when a rotorcraft enters a steep, high-rate descent [2]. The intense, unsteady vortex shedding induces violent low-frequency fluctuations in blade loading (as shown in Figure 1), which traditional momentum theory fails to model effectively [8].

To avoid this hazardous regime, the aviation industry predominantly employs threshold-based detection bounded by flight parameter envelopes [3]. Nevertheless, this threshold approach exhibits notable lag. By the time an IMU registers pitch and roll anomalies, the toroidal vortex structure has already fully formed [2]. Inherently serving as post-occurrence confirmation, traditional mechanisms are fundamentally incapable of executing proactive precursor early warning.

### 2.2. Data-Driven State Identification and the “Data Leakage” Crisis

To transcend the limitations of traditional dynamic models, deep learning techniques (CNNs, LSTMs) have been deployed to process fused time-series data from multi-source sensors [9]. However, applying these models to dynamic aviation scenarios often leads to a critical flaw: data leakage.

Because adjacent time slices of a continuous physical event are highly correlated, random splitting inevitably distributes highly similar temporal segments into both the training and testing sets [10]. Consequently, the model merely memorizes the background noise of a specific sortie rather than generalized aerodynamic fault features [5]. To eradicate this phenomenon, this study enforces a strict, physically isolated “sortie-based” dataset partitioning strategy, ensuring complete independence between training and blind-test flights [11].

### 2.3. Handling Extreme Imbalance: From Resampling to Ordinal Regression

Within a normal UAV flight log, the 1.5 s precursor window defined in this study accounts for a microscopic fraction of the data (typically <0.5%) [12]. Traditional classification networks optimized via standard Cross-Entropy are rapidly dominated by the majority class, rendering the model effectively blind to the rare precursor state [13].

Traditional classification treats “Normal,” “Precursor,” and “VRS” as independent categories, which is aerodynamically illogical. Drawing inspiration from disease progression monitoring, researchers have introduced Ordinal Regression concepts to maintain the continuity of the feature space [14]. This study creatively introduces a Hybrid Ordinal Loss into UAV state identification, leveraging physical continuity to combat extreme sample imbalance [15].

## 3. Materials and Methods

### 3.1. Experimental Platform and Avionics System

The data were collected using a customized coaxial dual-rotor UAV (max takeoff weight: 10 kg). The propulsion system, encompassing the rotors, electric motors, and electronic speed controllers (ESCs), was fully custom-designed and fabricated in-house to meet specific aerodynamic requirements. The rotor system operates at a rated speed of 2500 RPM, which serves as the physical reference for our subsequent sensor reliability analysis. To capture high-frequency dynamic responses, the UAV is equipped with a CUAV v5 flight controller (CUAV Tech Inc., Guangzhou, China). The multi-sensor payload comprises a BMI088 6-axis Inertial Measurement Unit (IMU) (Bosch Sensortec GmbH, Reutlingen, Germany) that logs 3-axis acceleration and angular velocity at 1200 Hz, an MS5611 high-resolution barometer (TE Connectivity, Schaffhausen, Switzerland) operating at 100 Hz, and a u-blox M8N GPS module (u-blox AG, Thalwil, Switzerland) functioning at 10 Hz. Detailed hardware specifications are summarized in Table 1.

All subsequent aerodynamic data extraction and dynamic modeling were processed utilizing Python v3.10 (Python Software Foundation, Wilmington, DE, USA) and MATLAB R2021a (The MathWorks Inc., Natick, MA, USA).

### 3.2. Multi-Source Cross-Validated Data Labeling

The quality of data annotation fundamentally dictates the theoretical performance limit of the predictive model. To ensure maximum confidence, this study replaces simplistic threshold-based labeling with a rigorous protocol that integrates flight test telemetry, CFD simulations, and scaled wind tunnel validations.

1. Kinematic Screening: GPS data isolates flight segments satisfying VRS onset conditions [8].
2. CFD Verification: Boundary conditions are extracted to conduct high-fidelity, unsteady flow field simulations [16]. A segment is confirmed only if it exhibits classic VRS topology.
3. Wind Tunnel Calibration: Scaled-model tests acquire empirical aerodynamic load data [17].
4. High-Precision Annotation: The raw flight onboard-recorded sensor data is rigorously annotated into classes.

**Figure 2:**
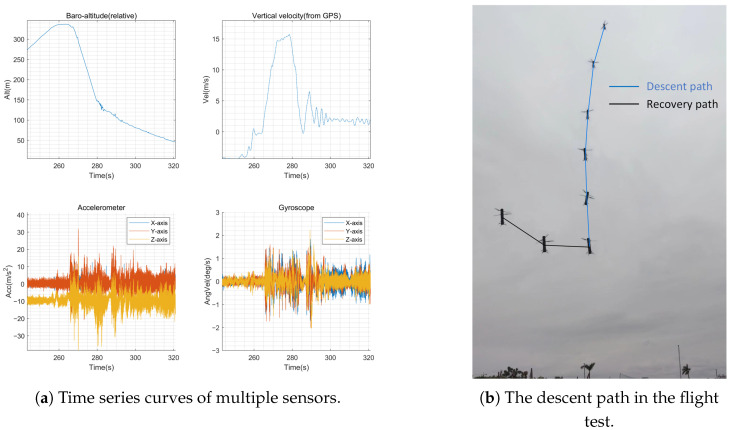
(**a**) A segment of the time-series curves from a flight test; (**b**) the descent path.

A representative segment of the raw multi-sensor telemetry data and the corresponding descent trajectory during the flight test are illustrated in Figure 2.

The statistical analysis (Table 2) shows that VRS samples represent a distinctly minor fraction, peaking at merely 7.78%.

### 3.3. Multimodal Time-Frequency Transformation

**Figure 3:**
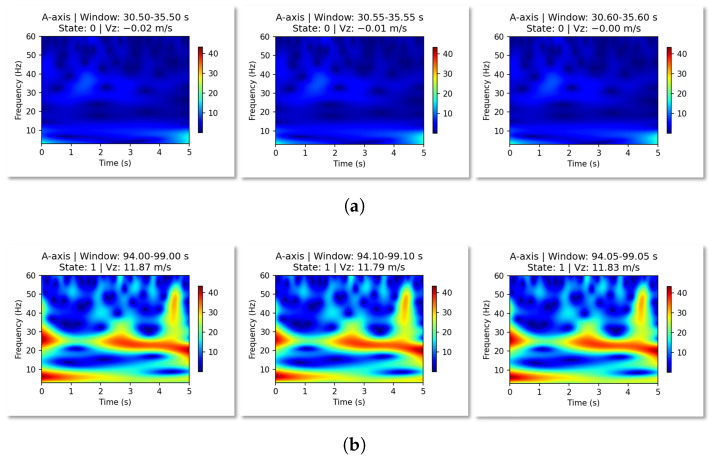
CWT time-frequency plot example. (**a**) Hovering stage; (**b**) high-speed descent.

To fundamentally eradicate “data leakage,” this study enforces a sortie-based protocol: training on Sorties 1–3, validation on Sortie 4, and blind testing exclusively on Sortie 5 [11]. The Continuous Wavelet Transform (CWT) with a Complex Morlet wavelet extracts high-resolution time-frequency representations [18], as shown in Figure 3. Transforming raw 1D vibration signals into 2D CWT spectrograms allows the architecture to simultaneously capture macroscopic flight trends in low-frequency regions and microscopic periodic signatures (e.g., the 1P harmonic) in high-frequency regions. While CWT introduces minor computational overhead, modern embedded computing platforms execute this at the millisecond scale, remaining fully compatible with the 1.5 s early warning window.

#### Engineering Causality Constraint

To ensure the algorithm can perform zero-lookahead, unidirectional real-time inference within a flight control system, we enforce a strict engineering causality constraint. The CWT is computed via a strictly causal sliding filter bank with a one-sided temporal support, ensuring no future samples are accessed. A sliding window of 5 s is employed, where an asymmetric Hanning boundary weighting is applied to unequivocally sever the reliance on any forward-looking samples.

### 3.4. Calibrated Benchmark Normalization (CBN) Strategy

This study proposes an innovative Calibrated Benchmark Normalization (CBN) strategy to embed physical aerodynamic scales into the data preprocessing pipeline. This physical anchoring ensures that the amplitude disparities between low-vibration nominal states and turbulent states are preserved, unlike standard local batch normalizations that destruct such absolute scales.

1. Offline Calibration: Extracting the 0.1th percentile (Hover state) as ${\mathrm{Calib}}_{\mathrm{Min}}$ and the 99.9th percentile (Windmill state, representing the unpowered autorotation regime where the rotors freewheel, marking the aerodynamic upper bound of vibration) as ${\mathrm{Calib}}_{\mathrm{Max}}$. Importantly, these calibration percentiles are derived exclusively from offline calibration flights; absolutely no data from the test set (Sortie 5) is utilized during this phase.
2. Deployment Phase:
  (1) $$X_{\mathrm{norm},c}=\mathrm{clip}\left(\frac{X_{c}-{\mathrm{Calib}}_{\mathrm{Min},c}}{{\mathrm{Calib}}_{\mathrm{Max},c}-{\mathrm{Calib}}_{\mathrm{Min},c}},\;0,\;1\right)$$

This decoupled computation strictly fulfills real-time processing constraints.

The 0.1th and 99.9th percentiles were deliberately adopted instead of absolute extrema (0th and 100th) to filter out impulsive high-frequency sensor anomalies and mechanical shocks, ensuring that the normalization bounds represent genuine aerodynamic steady states. The normalization scale is inherently robust to these threshold selections, as the core physical energy distributions of the Hover and Windmill states are statistically concentrated.

### 3.5. Proactive Warning Strategy and Hybrid Ordinal Loss

To prevent the generation of highly redundant sliding windows, we adopt an event-based extraction strategy for the testing phase. Specifically, only the discrete 1.5 s window immediately preceding a fully developed VRS event is extracted as a single distinct “Precursor” instance. Consequently, the blind-test sortie yields 28 distinct precursor events corresponding to 28 impending VRS events. The same event-based extraction strategy is applied consistently to the training phases to ensure distribution alignment, yielding a total of 146 precursor instances across Sorties 1–4.

We retrospectively relabeled the final 1.5 s preceding a fully developed VRS as an independent “Precursor” class. To counteract severe class imbalance (<0.5%), we introduce a Hybrid Ordinal Loss [15]:

(2) $$L_{\mathrm{hybrid}}=\alpha L_{\mathrm{WCE}}+\beta L_{\mathrm{MSE}}$$

where α regulates the fundamental classification capability of the Weighted Cross-Entropy (WCE) component to address sample scarcity, and β controls the ordinal aerodynamic degradation penalty via the expectation-based MSE component. In this formulation, α is fixed at 1.0 to establish the WCE as a standardized baseline classification anchor. Tuning only β systematically evaluates the relative regulatory strength of the physical ordinal constraint without expanding the optimizer’s redundant scaling search space.

The MSE component imposes an elastic physical constraint:

(3) $$L_{\mathrm{MSE}}={\left(\sum_{i=1}^{C}i\;\cdot \;{\hat{y}}_{i}-\sum_{i=1}^{C}i\;\cdot \;y_{i}\right)}^{2}$$

This explicitly forces the network to respect the irreversible degradation direction of the aerodynamic state [19].

### 3.6. The MTSF-Net Early Warning Architecture

**Figure 4:**
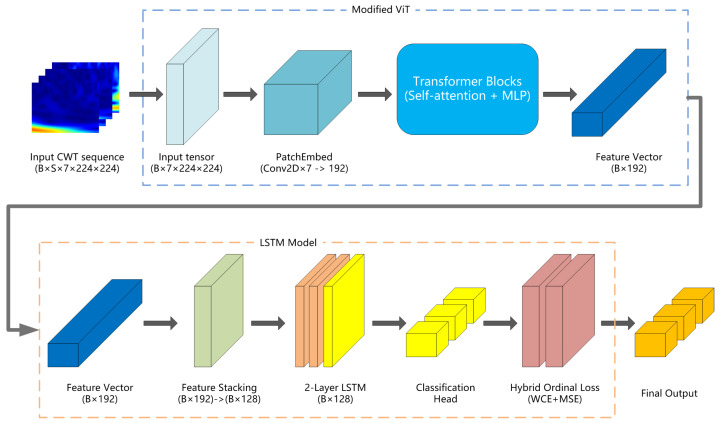
MTSF-Net network topology including ViT and LSTM backbones.

The MTSF-Net processes seven flight control channels (3-axis Accl, 3-axis Gyro, and $V_{z}$). Following CWT and CBN, the 2D spectrograms generate a multi-channel tensor. The Vision Transformer (ViT) acts as the spatial-frequency feature extractor [20], feeding its output sequence into a two-layer stacked LSTM network for temporal evolution modeling. The final layer generates 3-class ordinal logits. The network topology of MTSF-Net is shown in Figure 4.

## 4. Results

### 4.1. Ablation Study on Architecture and Modality

To rigorously validate the efficacy of the core components within the proposed MTSF-Net, a comprehensive ablation study was conducted on the strictly quarantined, unseen test set (Sortie 5). The ablation results are illustrated in Table 3.

The decisive role of multimodal fusion is proven by comparing Baselines A through D. Baseline A struggles to capture early degradation. Adding gyroscope data (Baseline B) or vertical velocity (Baseline C) incrementally improves performance, but it is the full seven-channel integration in Baseline D that provides a comprehensive state representation. Furthermore, replacing traditional local batch normalization with the physically anchored Calibrated Benchmark Normalization (CBN) in the proposed MTSF-Net triggers a remarkable surge in Precursor recall. Note that the MTSF-Net in this ablation study adopts the baseline Hybrid Ordinal Loss hyperparameters (α=1.0,β=1.0). Further hyperparameter optimization to break the performance bottleneck is detailed in Section 4.2.

### 4.2. Sensitivity Analysis of Hybrid Ordinal Loss Hyperparameters

To rigorously justify the selection of the hyperparameters α and β in the proposed Hybrid Ordinal Loss (Equation (2)), and to demonstrate their impact on model reproducibility and class balance, a comprehensive sensitivity analysis was conducted. The parameter α was fixed at 1.0 to ensure the foundational classification capability provided by the Weighted Cross-Entropy. We systematically evaluated the ordinal penalty weight β across a range of values β∈[0.0,0.5,0.85,1.0,1.15,1.3,1.5] on the unseen test set (Sortie 5). Notably, the configuration β=0.0 represents Scheme A, where the model is optimized solely by standard Weighted Cross-Entropy without any ordinal physical constraints.

As summarized in Table 4, the model’s performance exhibits high sensitivity to the ordinal penalty weight. The configuration β=1.15 emerges as the optimal balance point. At this value, the model achieves the highest test accuracy (98.26%) and the lowest Mean Absolute Error (MAE = 0.0290). Most crucially, for the early warning task, β=1.15 successfully rescues the critically scarce Precursor class, elevating its F1-Score to 0.789 while maintaining highly robust VRS detection (F1-Score = 0.877).

Conversely, entirely removing the ordinal constraint (β=0.0, Scheme A) completely collapses the model’s overall accuracy to 9.63%. While the model is forced to achieve a deceptive 100% Precursor recall due to extreme class weighting, it sacrifices the Normal state accuracy (dropping to approx. 3%), resulting in massive false positives and a dismal Precursor F1-Score of 0.023. Furthermore, assigning insufficient weight to the ordinal constraint (e.g., β∈[0.5,0.85]) or excessively penalizing it (e.g., β≥1.3) leads to severe feature space distortion, causing the model to completely fail in recognizing the Precursor state (Precursor F1 drops to 0.000). This sensitivity analysis explicitly validates the theoretical necessity of the Hybrid Ordinal Loss and confirms α=1.0,β=1.15 as the optimal combination for real-world deployment.

From a feature representation perspective, the failure at suboptimal weights (β∈[0.85,1.30]) is not due to gradient vanishing, but rather feature space collapse driven by extreme class imbalance. At β=0.85 (under-penalized), the WCE dominates, causing the network to subsume the scarce Precursor minority into the Normal majority to maximize global accuracy. At β=1.30 (over-penalized), the rigid MSE penalty over-constrains the intermediate Precursor features, pulling them toward the well-defined boundaries of the dominant classes and collapsing their independent linear separability.

### 4.3. Early Warning Performance and the Necessity of Ordinal Constraints

Under the most rigorous cross-sortie test evaluation, MTSF-Net demonstrates exceptional performance. To rigorously validate the physical ordinal constraint, we compared the optimally configured MTSF-Net (β=1.15) against a baseline model optimized solely by standard Weighted Cross-Entropy, hereafter referred to as Scheme A.

**Figure 5:**
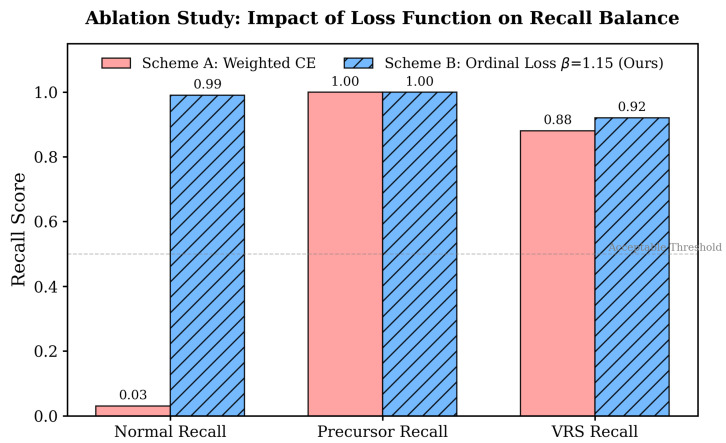
Recall balance comparison between Scheme A (standard WCE) and the proposed Hybrid Ordinal Loss (β=1.15).

As illustrated in Figure 5, relying exclusively on extreme class weighting (Scheme A) to combat the <0.5% precursor sample imbalance triggers significant performance degradation. Scheme A tends to severely over-predict the minority class to artificially inflate Precursor recall, causing the Normal state accuracy to plummet to a mere 3%. Such an exploding false-positive rate (FPR) would induce severe alarm fatigue, rendering the flight control system practically unusable.

**Figure 6:**
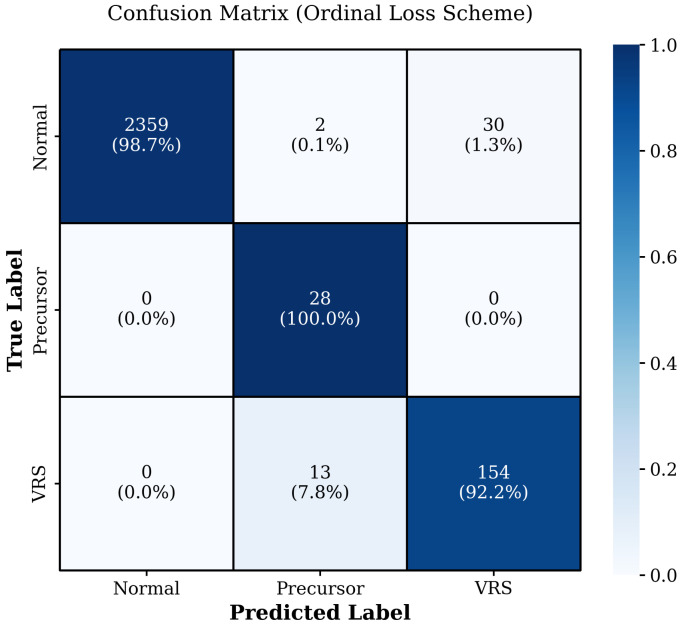
Confusion matrix of the MTSF-Net. It demonstrates the capability to maintain an exceptionally high true-negative rate while achieving robust proactive detection for Precursor and VRS states.

In stark contrast, the proposed Hybrid Ordinal Loss acts as a robust, physics-informed regularizer. By setting the optimal ordinal penalty weight (β=1.15), it effectively rescues the model from aggressive over-prediction while maintaining extreme sensitivity to hazardous states. As detailed in the confusion matrix (Figure 6), this constraint secures comprehensive breakthroughs:

- **Robust Nominal Flight Accuracy (98.6%):** The network successfully maintains a high true-negative rate for safe hovering and basic maneuvers, avoiding severe alarm fatigue.
- **Optimal VRS Detection (92.2%):** The model achieves robust identification of fully developed VRS hazard zones.
- **Proactive Precursor Interception (100.0%):** The model successfully intercepted all 28 impending VRS samples in the unseen test set, demonstrating an optimal capability to secure the crucial 1.5 s intervention window.
- **Elimination of Fatal Misclassifications:** Catastrophic cross-level misclassifications were completely suppressed. Fatal missed detections (VRS samples misclassified as Normal) were reduced to 0.0%, and false-positive VRS predictions triggered by Precursor states were also completely eliminated (0.0%).

## 5. Discussion

### 5.1. Verification of Sensing Interpretability via Grad-CAM

A critical requirement for deploying multi-sensor deep learning systems in aviation is the interpretability of the decision-making process. It is imperative to verify that the proposed MTSF-Net relies on authentic physical signals rather than environmental noise.

**Figure 7:**
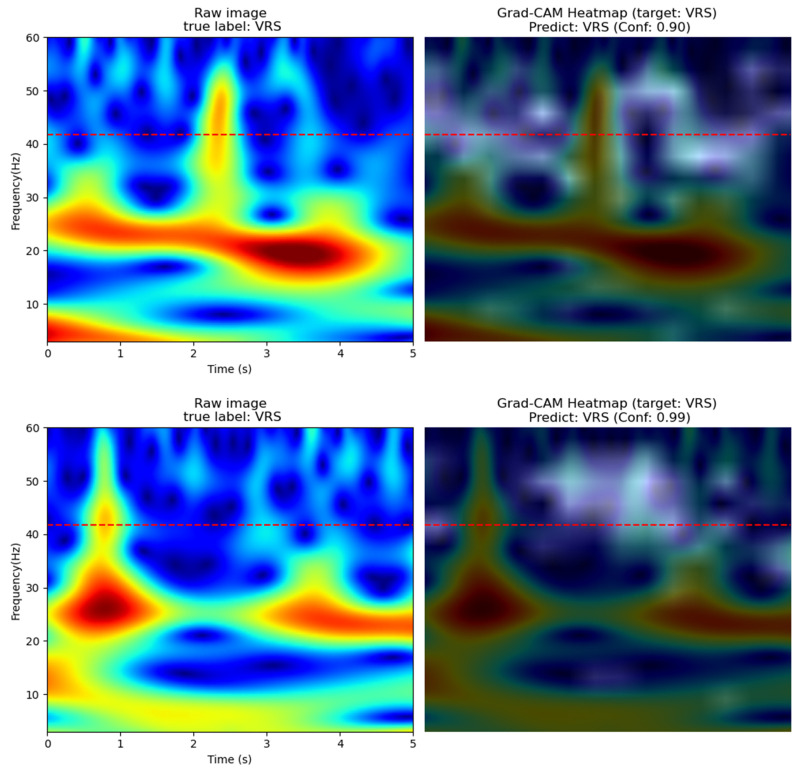
Physical interpretability validation via Gradient-weighted Class Activation Mapping (Grad-CAM). The heatmaps explicitly demonstrate that the network’s predictive attention is anchored to the specific 1P frequency (marked with the red dashed lines) band.

By extracting the Gradient-weighted Class Activation Mapping (Grad-CAM) heatmaps (Figure 7), a highly desirable phenomenon emerges. The attention weights of the Vision Transformer (ViT) encoder explicitly anchor onto a specific high-intensity energy band distributed between 30 Hz and 45 Hz. This visually quantifiable localization aligns with the theoretical first harmonic (1P) fundamental frequency (≈41.7 Hz) of the coaxial dual-rotor system’s aerodynamic vibration. This confirmation provides robust evidence that our seven-channel sensor fusion pipeline and CBN strategy successfully preserve and utilize genuine physical telemetry features.

### 5.2. Robustness and Algorithmic Conservatism: Analyzing False Negatives

**Figure 8:**
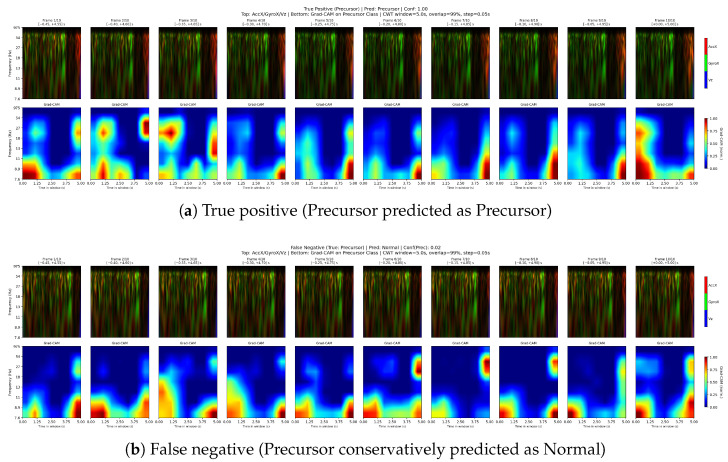
Spatiotemporal Grad-CAM visualization of MTSF-Net’s attention dynamics across a continuous 10-frame sequence during the incipient Precursor phase.

An in-depth analysis of the continuous spatiotemporal Grad-CAM visualizations (Figure 8) reveals a highly desirable engineering trait inherent in the proposed MTSF-Net: algorithmic conservatism. In true-positive (TP) instances (Figure 8a), the attention heatmaps exhibit strong, coherent, and highly localized tracking of critical aerodynamic frequency bursts over time.

Conversely, in false-negative (FN) instances (Figure 8b)—where a Precursor is conservatively predicted as Normal—the raw time-frequency features are typically heavily corrupted by environmental noise or aggressive maneuvers. Unable to establish a stable physical anchor, the Vision Transformer’s attention diffuses. Guided by the Hybrid Ordinal Loss, the model refuses to trigger a spurious alarm based on chaotic signals and defaults to the last known safe state, successfully avoiding alarm fatigue.

### 5.3. Engineering Implications: The Value of Early Warning

From a sensor-to-actuation system perspective, establishing a 1.5 s precursor early warning window holds substantial engineering utility. Traditional rigid-body sensors (such as purely measuring vertical descent rate) suffer from inherent physical latency. In contrast, the MTSF-Net extracts high-frequency structural vibration precursors directly from the IMU, completely bypassing this kinematic lag. Once the 1.5 s lead time is secured during the incipient Precursor phase, the flight computer can seamlessly issue differential RPM commands to the Electronic Speed Controllers (ESCs) at millisecond latency, actively breaking the aerodynamic symmetry of the vortex ring before the accumulation of fatal macroscopic descent velocity.

### 5.4. Limitations and Future Work

While the proposed MTSF-Net framework demonstrates exceptional proactive early warning capabilities, this study has certain limitations that warrant further investigation. First, the current dataset and the corresponding optimal hyperparameter configurations (α=1.0,β=1.15) were derived from a single customized coaxial dual-rotor UAV platform. Generalizing this physics-informed framework to rotorcraft with completely different aerodynamic configurations (e.g., conventional single-rotor or quadrotors) or significantly varying gross weights requires further cross-platform validation. Second, although the 1.5 s warning window provides sufficient reaction time, the inference pipeline has not yet been integrated with active flight control laws for closed-loop autonomous recovery testing in real-world extreme environments.

Future research will primarily focus on two directions. First, we aim to conduct comprehensive cross-platform flight tests to verify the robustness of the Calibrated Benchmark Normalization (CBN) strategy and the 1P fundamental frequency feature across diverse UAV configurations. Second, we will explore model compression and quantization techniques for the ViT-LSTM architecture to facilitate ultra-low-latency, edge-computing deployment on resource-constrained onboard flight controllers.

## 6. Conclusions

This study pioneers a closed-loop technological pipeline that bridges sensor telemetry with proactive deep learning condition monitoring, offering a new paradigm for Vortex Ring State (VRS) defense architectures in coaxial dual-rotor UAVs. The pioneering Calibrated Benchmark Normalization (CBN) strategy successfully embeds true physical aerodynamic extrema into the real-time inference pipeline. Coupled with the carefully tuned Hybrid Ordinal Loss, the framework decisively overcomes the optimization challenges induced by extremely imbalanced precursor data.

In the ultimate blind-test validation, the model achieved an exceptional overall test accuracy of 98.26% and a precursor recall of 100%. Crucially for aviation safety, the ordinal constraint mechanism completely eliminated fatal missed detections of the VRS state. From an engineering perspective, this study leveraged Grad-CAM XAI technology to verify that the AI’s predictive attention is precisely anchored onto the theoretically derived 41.7 Hz frequency band, validating that the model learned authentic aerodynamic mechanisms rather than spurious noise. These findings lay a robust, interpretable foundation for the future deployment of certifiable, proactive safety systems in autonomous aviation.

## Figures and Tables

**Table 1:** Sensor parameters of the UAV flight control system.

Sensor Type	Freq. (Hz)	Main Performance
Accelerometer	1200	Range: 200 G;
		Zero-bias Stability: 0.8 mg;
		Bandwidth: 200 Hz.
Gyroscope	1200	Range: 2000° s^−1^;
		Zero-bias Stability: 16° h^−1^;
		Bandwidth: 200 Hz.
Barometer	100	Working Pressure: 100–1300 hPa;
		Resolution: ± 0.002 hPa;
		Relative Accuracy: ± 0.06 hPa.
GPS	10	Horizontal Accuracy: 0.2 m;
		Elevation Accuracy: 0.5 m.

**Table 2:** Raw flight data statistics.

Sortie No.	IMU Samples	VRS Samples	VRS %
1	164,419	9855	5.99%
2	202,515	10,638	5.25%
3	224,192	11,515	5.14%
4	328,621	25,563	7.78%
5	324,447	17,824	5.49%

**Table 3:** Ablation study results on the unseen test set (Sortie 5).

Network	Input Sensor	Data Norm.	Global	Precursor	VRS
Configuration	Modalities	Strategy	F1-Score	Recall	Recall
Baseline A	Accelerometer (3 ch)	Local Batch Norm	76.2%	14.2%	81.5%
Baseline B	Accl. + Gyroscope (6 ch)	Local Batch Norm	82.5%	28.6%	85.1%
Baseline C	Accl. + $V_{z}$ (4 ch)	Local Batch Norm	84.1%	35.9%	88.3%
Baseline D	Full Multimodal (7 ch)	Local Batch Norm	88.7%	46.1%	90.2%
**MTSF-Net (Proposed)**	**Full Multimodal (7 ch)**	**Physical Calibrated CBN**	**94.4%**	**60.7%**	**94.0%**

**Table 4:** Sensitivity analysis of the ordinal penalty weight β (α=1.0). The configuration β=0.0 represents Scheme A (pure WCE). The optimal configuration (β=1.15) is highlighted in bold.

Weight (β)	Test Accuracy	MAE	Macro F1	Precursor F1	VRS F1
0.00 (Scheme A)	0.0963	0.9320	0.323	0.023	0.890
0.50	0.9269	0.0959	0.473	0.015	0.425
0.85	0.9625	0.0642	0.592	0.000	0.795
1.00 (Baseline)	0.9764	0.0379	0.818	0.586	0.880
**1.15 (Optimal)**	**0.9826**	**0.0290**	**0.886**	**0.789**	**0.877**
1.30	0.9687	0.0503	0.608	0.000	0.839
1.50	0.9551	0.0514	0.676	0.182	0.864

## Data Availability

The data presented in this study are available on request from the corresponding author. The data are not publicly available due to institutional restrictions and ongoing research.

## Abbreviations

1P	First Harmonic Fundamental Frequency
Accl	Accelerometer
CBN	Calibrated Benchmark Normalization
CFD	Computational Fluid Dynamics
CNN	Convolutional Neural Network
CWT	Continuous Wavelet Transform
ESC	Electronic Speed Controller
FN	False Negative
FPR	False-Positive Rate
GPS	Global Positioning System
Grad-CAM	Gradient-weighted Class Activation Mapping
IMU	Inertial Measurement Unit
LSTM	Long Short-Term Memory
MSE	Mean Squared Error
MTSF-Net	Multi-channel Time-Frequency Fusion Network
RPM	Revolutions Per Minute
TP	True Positive
UAV	Unmanned Aerial Vehicle
ViT	Vision Transformer
VRS	Vortex Ring State
WCE	Weighted Cross-Entropy
